# Transformation and analysis of tobacco plant var *Petit havana* with T-*ur*f13 gene under anther-specific TA29 promoter

**DOI:** 10.1007/s13205-011-0008-6

**Published:** 2011-06-01

**Authors:** V. Arun, Boney Kuriakose, Vaniyambadi V. Sridhar, George Thomas

**Affiliations:** 1 Department of Biotechnology FBMST, Sri Ramachandra University, Porur, Chennai 600116 India; 2 Department of Plant Sciences, Forest and Agricultural Biotechnology Institute, University of Pretoria, Pretoria, South Africa; 3 Department of Biological Sciences, University of New Orleans, New Orleans, USA; 4 Interfield Laboratories, Cochin, 682 005 India; 5 Spic Science Foundation, Guindy, Chennai 600032 India

**Keywords:** *cms*-T maize, Hybrid seeds, Male sterility, Tobacco transformation, T-*urf*13, URF13

## Abstract

T-*urf*13, a well-documented *cms*-associated gene from maize, has been shown to render methomyl sensitivity to heterologous systems like rice, yeast and bacteria when expressed constitutively. Since these transgenic plants were fertile, it was hypothesized that T-*urf*13 gene if expressed in anthers may result in male sterility that could be used for hybrid seed production. Hence, this work was aimed at analysing whether T-*urf*13 gene when expressed in anthers can result in male sterile plants or requires methomyl treatment to cause male sterility (controllable). This is the first report of transformation of tobacco with T-*urf*13 gene under anther-specific promoter (TA29) with or without mitochondrial targeting sequence. Most of the transgenic plants obtained were fertile; this was surprising as many male sterile plants were expected as T-*urf*13 gene is a *cms* associated gene. Our results suggest that it may not be possible to obtain male sterility by expressing URF13 in the anther by itself or by methomyl application.

## Introduction

The T-*urf*13 gene (*cms*-T) is a cytoplasmic male sterility (CMS)-associated gene that was exploited for hybrid seed production in maize during 1960s in the Southern United States (Levings 1990). The T-*urf*13 gene was first identified in *cms*-T maize plants and it rose via homologous recombination between 5′ region of *atp6* gene and 3′ region of *rrn*26 gene resulting in a functional 13 kDa protein, URF13 (Dewey et al. 1986; Sofi et al. 2007). The URF13 is unusual in that the majority (~88%) of its sequence is coded by 3′ coding region of *rrn*26 gene, a region that normally does not code for any protein (Pring and Lonsdale 1989). This protein is an integral membrane protein of the inner mitochondrial membrane of *cms*-T maize plants (Forde and Leaver 1980) and has been predicted to possess three membrane-spanning domains due to its highly amphoteric amino terminus. The functionality of this protein in *cms*-T maize plants was detected phenotypically as these plants were sterile (male) and also susceptible to fungal toxins (T and P) and methomyl. This gene is unique to *cms*-T maize plants and has been shown to cause male sterility in these plants (Dewey et al. 1987).

The exact mechanism by which URF13 causes male sterility in maize has not been elucidated (Budar et al*.*^2003^; Levings 1993; Schnable and Wise 1998; Sofi et al. 2007). The defects observed in *cms*-T anthers included degeneration of mitochondrial cristae of tapetal cells and disorganization of the middle layer after meiosis (Warmke and Lee 1977). Even though URF13 is expressed constitutively in *cms*-T maize plants, the mitochondria of the anthers alone showed morphological defects indicating tissue specificity of the cytotoxic effect of URF13. Two hypotheses were put forward to explain male sterility in *cms*-T maize: (1) Flavell (1974) hypothesized that in young maize anthers a biosynthetic product is produced that mimics the effects of methomyl or T-toxin and causes male sterility in *cms*-T maize (gain-of-function model) and (2) URF13 alters mitochondrial functions like respiration, permeability of inner mitochondrial membrane and alternate oxidase pathway in the *cms*-T maize plants. The effects were more pronounced in anthers of these plants and results in male sterility (loss-of-function model) (Budar et al. 2003; Connet and Hanson 1990).

Fertility is restored by the action of nuclear genes (*Rf*1, *Rf*2, *Rf*8 and Rf*). Recently, *Rf*2 has been identified as mitochondrial aldehyde dehydrogenase and restoration was sporophytic (i.e.) 100% fertile pollen (Budar et al. 2003; Levings 1990). Since the fertility restoration was nearly 100%, the *cms*-T maize plants were extensively used for hybrid production until 1970s. *cms*-T maize plants were sterile (male) due to functional URF13 in anthers even though this protein was expressed constitutively. Because of this the *cms*-T maize plants were highly susceptible to the toxins, T-toxin and P-toxin produced by fungal pathogens *Bipolarismaydis* and *Phyllostictamaydis,* respectively, and resulted in severe losses (Ullstrup 1972). The interaction of fungal toxin with URF13 in *cms*-T plants has been shown to inhibit mitochondrial respiration and malate-dependent electron transport system, uncoupling of oxidative phosphorylation, increased permeability of H^+^ and Ca^2+^ ions (Sofi et al. 2007). Interestingly a carbamate insecticide, methomyl (active ingredient of Lannate of DuPont) that does not have any structural similarity to the fungal toxins also had similar effects on these plants (Levings 1990). T-*urf*13 gene has been expressed in heterologous systems like yeast, insect cell cultures and *E. coli*. The functionality of the URF13 protein in these systems was demonstrated by their sensitivity to methomyl treatment. Transformed yeasts that had URF13 targeted to mitochondria were more susceptible to methomyl treatment than the ones where URF13 was not targeted (Glab et al*.*^1993^; Huang et al. 1990). Many of the transgenic tobacco carrying T-*urf*13 gene under constitutive promoter (CaMV35S) were sensitive to methomyl treatment but very few male sterile plants were obtained (~3–5%) (Chaumont et al. 1995). In our laboratory, rice was transformed with T-*urf*13 gene driven by *Ubiquitin* promoter with and without mitochondrial targeting sequence (*ATP9* subunit of *Neurospora crassa*). The mitochondria isolated from the rice that carried mitochondrial-targeted T-*urf*13 alone showed sensitivity to methomyl as confirmed by stimulation of NADH-driven respiration and inhibition of malate-stimulated electron transfer. Most of the transgenic rice were sensitive to methomyl treatment but were fertile (Sridhar 2001).

Expression of reporter genes like *GUS* and *GFP* with constitutive promoter, CaMV35S was observed in pollen but not in tapetum (sporogenous tissue of anther) (de Mesa et al. 2004; Plegt and Bino 1989). It was hypothesized that since the expression by *CaMV*35S and *Ubiquitin* promoters is less pronounced in reproductive tissues than in other tissues, transgenic plants expressing T-*urf*13 gene constitutively were fertile as URF13 protein levels might be less in anther tissues (Chaumont et al. 1995). Hence, it was proposed that if T-*urf*13 gene is expressed in anthers of plants, it could cause male sterility by itself (direct) or by methomyl application (induced male sterility) and hence could be used for hybrid seed production. We, therefore, attempted for the first time, transformation of tobacco with *T*-*urf*13 gene driven by anther-specific promoter (TA29 promoter, Accession number no X52283) for tissue-specific expression, with or without mitochondrial targeting sequence for male sterility.

## Materials and methods

### Plant materials and transformation

The tobacco (var *Petit havana*) seeds were surface sterilized using 2.5% hypo and germinated on ½ MS (Murashige and Skoog). Leaves from 1-month-old seedlings were used for *Agrobacterium*-mediated transformation (Horsch et al. 1985). The leaf discs were pre-cultured on full strength MS containing 30% sucrose (MS30) and after 2 days discs were co-cultivated with *Agrobacterium* LBA4404 for 1 min and incubated for 2 days on the same medium. The leaf discs were then washed with sterile water and selected on MS30 containing hygromycin (40 μg/ml). The calli obtained were subcultured every 21 days and regenerated transformed tobacco were rooted on ½ MS containing hygromycin (40 μg/ml) medium and transferred to green house. The inflorescences of the transgenic plants were bagged to prevent cross-pollination.

### Constructs used for tobacco transformation

**Fig. 1:**
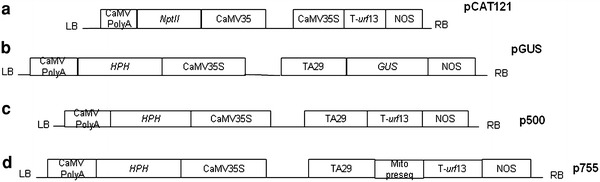
Schematic representation of the four constructs used in the study

All the constructs used were subcloned into pCAMBIA1200 backbone. Four constructs pGUS, pCAT121, p500 and p755 were used in the present study; of these pCAT121 and pGUS served as internal controls. The pCAT121 construct (Fig. 1a) where the T-*urf*13 gene was driven by CaMV35S promoter, was obtained by sub-cloning the T-*urf*13 gene from p500 into pSB301 (Bharadwaj 1999) as BamHI and SacI fragment. This construct was used to assay the sensitivity of the transgenic plants for methomyl treatment. Since the objective was to express the T-*urf*13 gene in anthers of tobacco, TA29 promoter, obtained using PCR, was tested for anther-specific expression using *GUS* gene (pGUS, Fig. 1b). The T-*urf*13 gene (500 bp) was subcloned from pEMBLYe30/2-21 URF13-TW, which was a gift from Dr. Nathalie Glab, was cloned with (p755 construct) and without (p500 construct) mitochondrial targeting sequence (ATP synthase subunit 9 of *Neurosporacrassa*, Fig. 1c, d). All the clones obtained were sequenced and mobilized into *Agrobacterium* LBA4404 by tri-parental mating (Ditta et al. 1980) and transformed *Agrobacterium* were confirmed by PCR for the presence of plasmid before using for tobacco transformation.

### Molecular analysis of transgenic plants

Genomic DNA was isolated from transgenic plants (Dellaporta et al. 1983) and PCR was done using specific primers to screen the putative transgenic plants obtained. PCR Southern analysis was done for some of the transgenic plants carrying p500 and p755 constructs. Briefly, PCR was done using TA29 forward and NOS reverse primers and the conditions were 94 °C for 5 min, followed by 27 cycles of 94 °C- 45 s, 51 °C- 1 min, 72 °C- 1.3 min and final extension of 72 °C for 10 min. The PCR products were diluted 1:50 and one part was separated on agarose gel and blotted onto N^+^ nylon membrane and probed with radio-labelled probe T-*urf*13 gene obtained by digesting the p500 construct with BamHI and SacI enzymes. The probed blot was exposed to X-ray for 30 min and developed. Southern analysis (Sambrook et al. 1989) was carried out, for transgenic plants carrying p500 and p755 constructs, with 20 μg of genomic DNA digested with HindIII and SacI restriction enzymes to show the presence of the gene. The digested DNA was separated on 1% agarose gel and transferred onto N^+^ nylon membrane by alkaline transfer. The nylon membrane carrying digested DNA was then probed at 56 °C with the 1.1 kb radio-labelled fragment (TA29 promoter and T-*urf*13 gene obtained by digestion of p500 with HindIII and SacI enzymes). The membrane was then sequentially washed twice with 3X and 1X SSC along with 0.1% SDS at 56 °C followed by exposure to X-ray film for 4 days at −70 °C.

Northern analysis was done with transgenic plants that carried pCAT121 construct. Total RNA was isolated from leaves according to Chomczynski and Sacchi (1987) method with slight modifications. The extracted RNA was then blotted onto Nylon N^+^ membrane by neutral transfer using 20X SSC buffer. The blot carrying RNA from control (untransformed tobacco) and five transgenic plants carrying pCAT121 construct were probed with T-*urf*13 gene (500 bp), obtained by digestion of pCAT121 with BamHI and SacI restriction enzymes, at 65 °C overnight. The blot was then washed twice with 2X, 1X and 0.5X SSC along with 0.1% SDS at 65 °C for 15 min followed by exposure to X-ray film for 1 day at −70 °C.

### Methomyl sensitivity of transgenic plants carrying T-*urf*13 gene

Transgenic plants (nodal explants) carrying T-*urf*13 gene driven by CaMV35S promoter (pCAT121) were tested for methomyl sensitivity in ½ MS supplemented with 0.1, 0.2 and 0.3% of water-soluble methomyl (Methomyl 40% SP, DuPont INDIA) for 10–15 days. One untransformed plant was included as negative control for all three concentrations of methomyl. Interaction of URF13 protein expressed in transgenic plants carrying pCAT121 construct with methomyl would result in growth retardation due to necrosis. The methomyl sensitivity of the plants (treated with 0.1%methomyl) was assayed by modified Evan’s blue cell death method (Sridhar 2001). Briefly, 50 mg of tissue was treated with 1 ml of 0.25% Evan’s blue dye for 15 min and destained. Then the dye bound to dead cells was eluted by incubating tissues in 50% methanol and 1% SDS for 30 min at 50 °C and absorbance was read at 600 nm.

### Anther morphology and pollen germination analysis

Anthers of Stage 2 (Koltunow et al. 1990) of tobacco were used for paraffin embedded microtome sectioning. The transverse sections were then stained with Toluidine blue (0.5%) and morphological features were observed. In case of transgenic plants carrying *GUS* gene (pGUS), anthers were washed in phosphate buffer with 1% Triton X-100 for 1 h at 37 °C and hand sections taken using pithium were used for histochemical staining (Jefferson et al. 1987). The anther sections were soaked in GUS staining solution [X-Gluc (1 mM) in phosphate buffer (40 mM) and methanol 20%] and incubated at 37 °C for 16 h. Pollen was collected from transgenic plants carrying T-*urf*13 gene (p500 and p755) directly into modified pollen germination medium [Sucrose–15%, Ca(NO_3_)_2_–0.03% and Boric acid (pH 7)–0.01%] (Sridhar 2001), from just dehisced anthers of a single flower and three replicates were used to calculate pollen germination percentage.

## Results

### Anther-specific expression of *GUS* gene (pGUS construct)

**Fig. 2:**
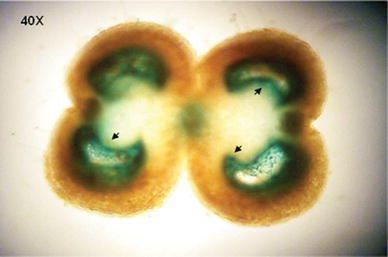
Transverse section of anther of transgenic tobacco plant (Gus-5) that carried pGUS construct showing expression of GUS (*black arrow heads*) in tapetum layer of the anthers

About 20 putative transgenic plants were obtained for pGUS and most of the plants (19 plants) were PCR positive (data not shown). These transgenic plants when tested for β-glucuronidase activity showed *GUS* was expressed in the anthers from Stage 2 (Fig. 2), and not in other parts of the plants; the other parts were also treated for GUS staining similarly (data not shown).

### T-*urf*13 gene constructs

#### Constitutive expression of T-*urf*13 gene (pCAT121 construct)

##### Molecular analysis

**Fig. 3:**
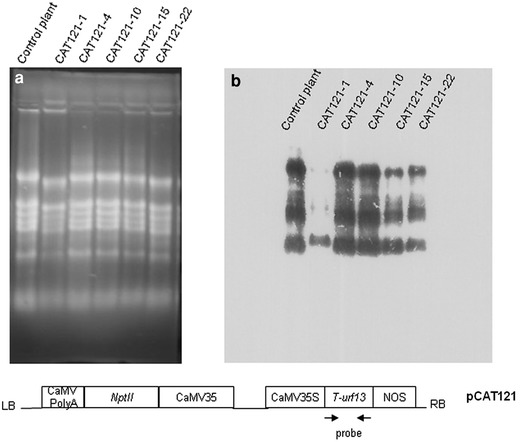
Northern analysis: **a** The RNA samples prior to blotting were shown; **b** Northern analysis of transgenic tobacco plants carrying pCAT121 construct. T-*urf*13 gene was used as probe that was obtained by digesting p500 with *Bam*HI and *Sac*I enzymes

**Fig. 4:**
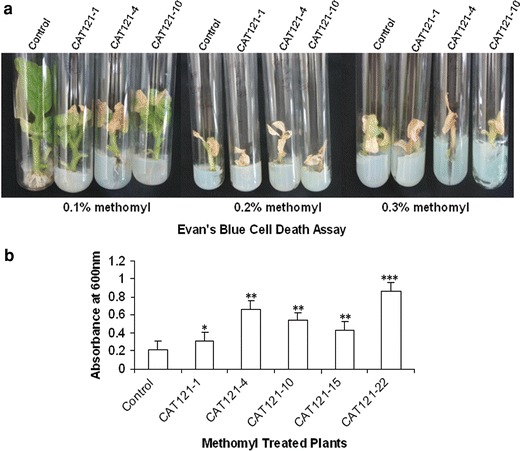
**a** Methomyl sensitivity of few tobacco plants transformed with T-*urf*13 gene driven by CaMV35S promoter (pCAT1121 construct); **b** The transgenic plants showed varied levels of methomyl sensitivity as evidenced by Evan’s cell death assay. The values obtained for Evan’s cell death assay were statistically significant (Student’s *t* test) and graph shows standard *error bars*

Around 15 PCR-positive putative transgenic plants carrying pCAT121 construct were obtained. Northern analysis was carried out for these transgenic plants using T-*urf*13 gene as probe. Since the probe (T-*urf*13 gene) has more than 80% homology to *rrn*26 gene it picked up most of the RNAs related to 26S rRNA (Fig. 3) (Pring and Lonsdale 1989). Three transgenic plants along with one control (untransformed) plant, in triplicates, were used for methomyl treatment. The transgenic plants carrying T-*urf*13 gene (pCAT121) showed varied degrees of growth retardation (cell death) due to interaction of URF13 with methomyl. Transgenic plants grown on 0.1% methomyl showed marked reduction in growth than control plant, whereas at higher concentrations even the control plants had reduced growth due to cell death (Fig. 4a). The cell death in these plants was assayed using Evan’s Blue assay where Evan’s blue dye binds only to dead cells. It was evident from the graph that cell death was significant in transgenic plants than in the control plants that were treated with methomyl (Fig. 4b). Evan’s blue assay also confirmed the growth retardation observed in transgenic plants carrying T-*urf*13 gene was due to cell death because of the interaction of URF13 and methomyl.

#### Anther-specific expression of T-*urf*13 gene (p500 and p755) in tobacco

##### Molecular analysis

**Fig. 5:**
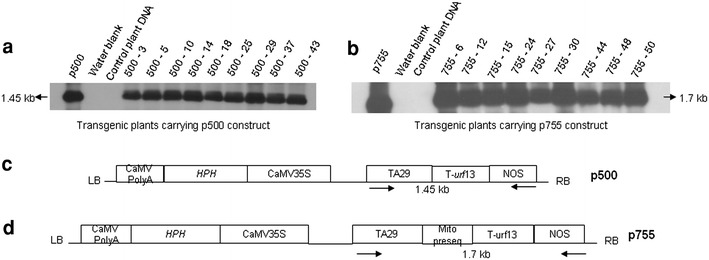
PCR Southern analysis of tobacco plants transformed with T-u*rf*13 gene constructs (p755 and p500). The PCR was done using TA29 forward and NOS reverse. The probe used was T-*urf*13 gene (~500 bp) and both the blots were probed separately. The expected band were ~1.45 kb for p500 and ~1.7 kb for p755 constructs as shown in (**c**, **d**). The absence of bands in the negative controls [water blank and control plant DNA (untransformed tobacco)] confirmed that the amplicons observed in lanes carrying DNA of transgenic plants were specific (**a**, **b**)

Presence of mitochondrial targeting sequence has been shown to enrich presence of URF13 protein in mitochondrial membrane in transgenic tobacco (Chaumont et al. 1995). Hence two constructs one without (p500 construct) and another with mitochondrial targeting sequence (p755 construct) were used in the present study (Fig. 1c, d). About 30 independent putative transgenic plants were obtained for each construct (p500 and p755) and analysed using PCR with gene-specific primers for both left and right borders of the cassette. Most of the putative transgenic plants were positive for presence of the gene (T-*urf*13, data not shown). PCR Southern analysis was done for nine transgenic plants for each construct (p500 and p755) along with negative controls (water blank and untransformed tobacco DNA). The PCR Southern showed the presence of expected amplicons of ~1.45 kb for p500 construct and ~1.7 kb for p755 construct (Fig. 5a, b). The negative lanes did not carry any amplicons (Fig. 5).

**Fig. 6:**
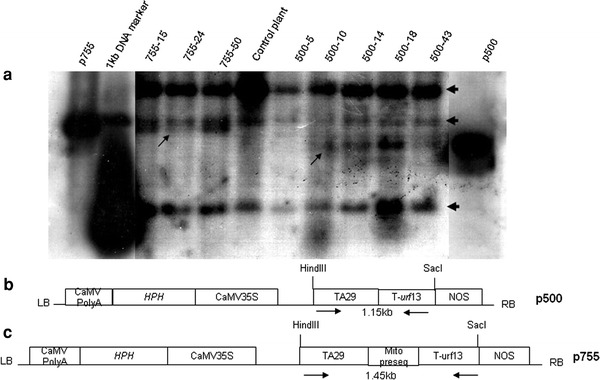
Southern analysis of tobacco plants transformed with T-u*rf*13 gene constructs (p755 and p500). The probe (~1.15 kb, consisted of TA29 promoter and T-*urf*13 gene) showed the presence of expected bands of ~1.15 and ~1.4 kb (*thin arrows*) for p500 and p755 constructs, respectively, and also had non-specific bands (corresponding to native *rrn*26 gene and TA29 promoter,*black arrows*)

Southern analysis was carried out using three putative transgenic plants carrying p755 construct (755-15, 755-24 and 755-50) and five putative transgenic plants carrying p500 construct (500-5, 500-10, 500-14, 500-18 and 500-43) that were positive for PCR. The genomic DNA was digested with HindIII and SacI enzymes that would release the TA29 promoter along with T-*urf*13 gene from p500 construct and in case of p755 construct the mitochondrial target sequence would also be released (Fig. 6b, c). The probe used was a fragment of 1.15 kb obtained by digestion of p500 with HindIII and SacI that consisted of TA29 promoter and T-*urf*13 gene. The expected fragment of ~1.15 kb and ~1.4 kb for p500 and p755, respectively, was observed in most of the transgenic plants (Fig. 6a). Few non-specific bands were also observed that corresponded to native TA29 promoter and also that of *rrn*26 gene (as T-*urf*13 has homology to *rrn*26 gene) as they were observed in untransformed tobacco lane also (Fig. 6a).

### Anther morphology and pollen germination

**Fig. 7:**
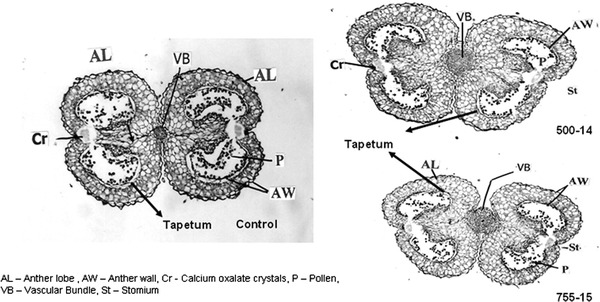
Comparison of transverse sections of anthers of control and transgenic plants carrying T-*urf*13 gene (755-15 and 500-14). The anther morphology appeared to be similar to that of control (untransformed tobacco) plant (i.e.) the URF13 protein did not affect the anthers of transgenic plants

**Fig. 8:**
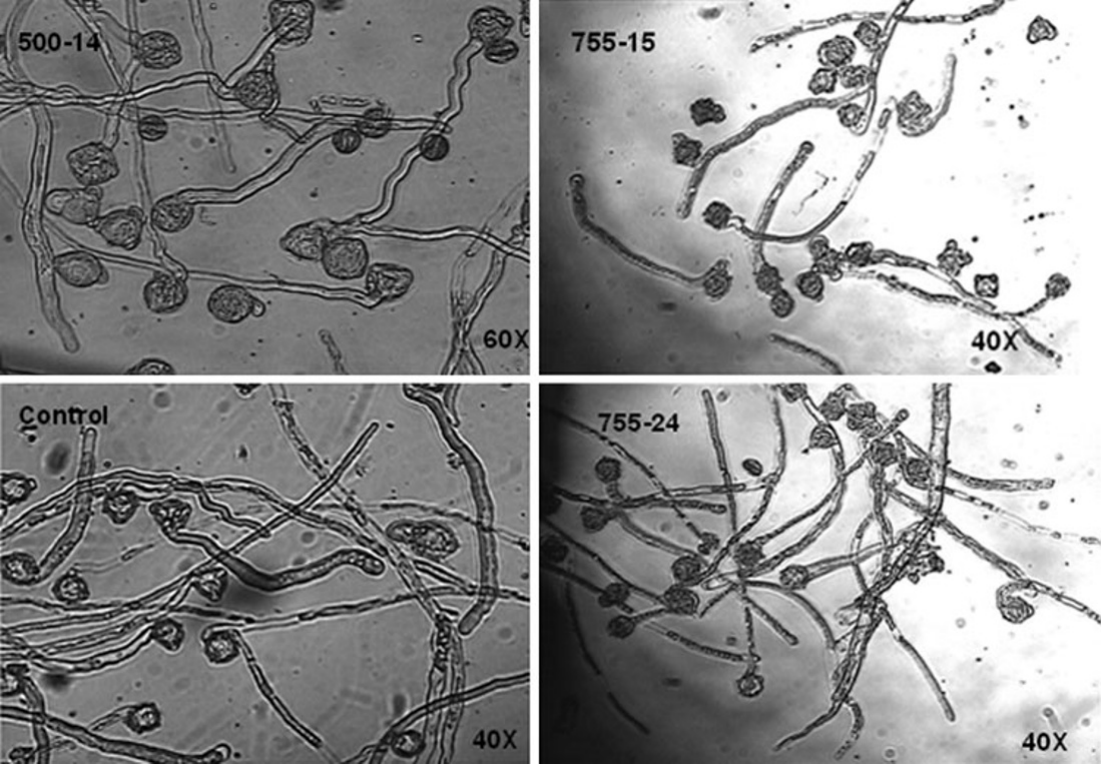
Pollen germination of transgenic plants (500-14, 755-15 and 755-24) and untransformed tobacco plant (control) were analysed. The pollen germination in the transgenic plants was normal when compared with untransformed tobacco plant

Most of the 30 transgenic plants obtained were fertile except 500-20 (that carried p500 construct, without mitochondrial targeting sequence) that was male sterile. This was surprising in that more number of male sterile plants was expected as it was hypothesized that if T-*urf*13 gene was expressed in anthers it might result in male sterility (Chaumont et al. 1995; Levings 1993; Sridhar 2001). The plant 500-20 had normal plant morphology but had severe reduction in pollen production. It was able to produce seeds when cross pollinated with pollen from control plants. Since majority of the transgenic plants obtained produced pollen only few plants were studied for anther morphology. The anther morphology of two transgenic plants (500-14 and 755-15) was similar to that of control anther (i.e.) tapetum layer was intact and pollen production also appeared normal (Fig. 7). The pollen germination analysis was done for three transgenic plants (500-14, 744-15 and 755-24) and it was observed that the pollen germination percentage was very similar to that of control plant (Fig. 8).

### Methomyl sensitivity of the transgenic plants

Methomyl treatment was done to evaluate the possibility of obtaining controllable male sterility in these transgenic plants that had T-*urf*13 gene (p500 and p755) by application of methomyl. The transgenic plants (PCR Southern and Southern positive, Figs. 5, 6) that carried p500 (5 plants) and p755 (3 plants) constructs along with one control tobacco (untransformed) plant were treated with three concentrations of methomyl (0.1, 0.2 and 0.3%) in the green house. The methomyl treatment was done with plants that had inflorescence with anthers between Stage 1–3 of development as TA29 has highest expression at Stage 2 (Koltunow et al. 1990).

Methomyl was administered to these plants by (1) irrigating with water, (2) spraying the inflorescence and (3) floral dipping along with Silwet L-77. The transgenic plants treated with methomyl produced viable pollen and seeds. All the three routes of methomyl administration had similar results (data not shown). This confirmed that the methomyl treatment had no effect on the anthers of these transgenic plants (p500 and p755 constructs) irrespective of mitochondrial targeting, since interaction of methomyl with URF13 protein (expressed in the anthers) would have resulted in tapetal degeneration and male sterility.

## Discussion

### GUS construct

The TA29 promoter has strong expression in the tapetum of tobacco anthers, and hence this promoter was chosen for the current study (Koltunow et al. 1990). The *GUS* expression was studied using Stage 2 anthers and showed good activity (Koltunow et al. 1990). The GUS activity was observed only in the tapetum and not in other parts of plant; this confirmed that the promoter had anther-specific activity and had no leaky expression. The transgenic plants had a normal morphology and produced viable seeds; this confirmed that the transformation procedure did not affect the plant morphology.

### T-*urf*13 constructs

#### Constitutive expression of T-*urf*13 gene (pCAT121 construct)

This construct was used to demonstrate the methomyl sensitivity of the transgenic tobacco plants expressing T-*urf*13 gene in a constitutive manner. PCR analysis confirmed that the putative transgenic plants carried the T-*urf*13 gene, and the presence of many bands in northern analysis confirmed that it is very difficult to differentiate the mRNA of T-*urf*13 from that of *rrn*26 owing to their homology (more than 80%). Also the *rrn*26 is one of the most abundant RNAs, and hence it would be very difficult to differentiate the mRNA of T-*urf*13 gene from that of *rrn*26 in these transgenic tobacco plants. The cell death observed in these transgenic tobacco plants upon treatment with methomyl is due to the interaction of URF13 with methomyl resulting in loss of mitochondrial membrane stability. Interaction of methomyl and URF13 in transgenic plants carrying T*urf*13 gene has been shown to cause cell death in those plants (Chaumont et al. 1995; Sridhar 2001; von Allmen et al. 1991).

The varied levels of sensitivity of transgenic plants to methomyl treatment confirmed the expression of URF13 protein in those plants and also confirmed the interaction of methomyl with URF13 protein. It was found that methomyl concentration 0.1% was enough to cause cell death in transgenic plants (Fig. 4a), indicating that T-*urf*13 gene was functional in these transgenic plants. The transgenic plants had a normal morphology and produced viable seeds which indicated that the constitutive expression URF13 in these plants had no effect on the anthers or seed production of these plants. The transgenic tobacco plants carrying T-*urf*`13 gene and *gus* gene also served as transformation control: most of the transgenic plants were fertile which confirmed that the transformation protocol worked well.

#### Anther-specific expression of T-*urf*13 gene (p500 and p755 constructs)

Many transgenic plants carrying p500 and p755 construct were obtained and analysed for the presence of transgene. PCR Southern analysis of transgenic plants carrying p500 and p755 construct showed that the amplicons obtained in PCR were highly specific. The PCR was carried out using TA29 forward and NOS reverse so that the amplicon would represent the right border consisting of the transgene (T-*urf*13) along with the promoter and terminator (Fig. 5c, d). The absence of any other amplicons in PCR Southern analysis further confirmed that the amplicon obtained were expected not spurious products. Southern analysis showed that the T-*urf*13 gene got integrated into the plant genome and though few non-specific bands were observed corresponding to native TA29 promoter and *rrn26* as those bands was observed in control lane also (Fig. 6a). Northern analysis was not attempted for these transgenic plants as the northern analysis in the transgenic plants carrying pCAT121 construct (Fig. 3) with T-*urf*13 gene as probe picked most of rRNAs. Since the gene-specific primers of T-*urf*13 might also pick *rrn*26 genes as T-*urf*13 gene has 80% homology to *rrn*26 gene and also the *rrn*26 genes are one of the most abundant genes, it would be very difficult to quantify the mRNA of T-*urf*13 through qPCR. Also transgenic tobacco carrying p755 or p500 construct would express T-*urf*13 in the tapetum and URF13 would be either targeted to inner-mitochondrial membrane or tapetal cell membranes, respectively. Isolation of the mRNA or URF13 protein in large amounts from Stage 2 tapetal tissues for northern and western analyses would be very difficult, as tapetum is an ephemeral layer comprising few cells. Advanced techniques like *in situ* hybridization, laser capture dissection microscopy could be employed to study T-*urf*13 expression in these plants (Emmert-Buck et al. 1996; Kerk et al. 2003).

The presence of many fertile transgenic tobacco plants that had normal anther morphology was interesting and contrasting to that observed in *cms*-T maize. Since only one male sterile plant (500-20) was obtained, it was not studied further due to the low percentage of male sterile plants (Chaumont et al. 1995; Sridhar 2001; von Allmen et al. 1991). Similar low percentage of male sterile plants was obtained when T-*urf*13 gene was expressed constitutively and the male sterile plants were not further characterized (Chaumont et al. 1995; Sridhar 2001; von Allmen et al. 1991). In this study we have used two other constructs (pGUS and pCAT121) and obtained atleast 25 transgenic plants each and they were found to be fertile. Hence, the male sterility observed could be due to URF13 protein but has to be confirmed by either in situ hybridization or other sophisticated methods. Also large number of male sterile plants has to be obtained and analysed to confirm that male sterility was due to URF13.

The anthers of these transgenic tobacco did not have any defects like degeneration of mitochondrial cristae of tapetal cells and disorganization of the middle layer after meiosis that were observed in *cms*-T maize plants (Warmke and Lee 1977). These defects affected the tapetum layer and pollen production resulting in male sterility in *cms*-T maize plants. The fertility of these transgenic tobacco plants was also confirmed by pollen germination percentage and it was found to be similar to that of untransformed tobacco plant (data not shown). We propose that the fertility of the transgenic tobacco plants (carrying T-*urf*13 gene) observed in the current study could be that these plants had mild to moderate levels of expression of T-*urf*13 gene. The cause of male sterility in *cms*-T maize plants was attributed to URF13 and URF13 has been localized to the inner membrane of mitochondria of tapetum of *cms*-T maize (Hack et al. 1991; Levings 1993). This was further confirmed by crossing the *cms*-T maize with restorer lines that carried *Rf* genes (*Rf1, Rf2, Rf8* and Rf*). The hybrids obtained from these crosses were fertile as they had highly reduced levels of URF13 protein (Budar et al. 2003; Sofi et al. 2007).

Even though the male sterility in *cms*-T maize was attributed to URF13 protein, the exact mechanism by which it causes male sterility is still not understood (Schnable and Wise 1998). It has been hypothesized by Flavel (1974) that in the anthers of those maize plants a protein or metabolite that mimics the fungal T-toxin or methomyl (insecticide) is produced and interacts with URF13 thereby resulting in male sterility. Hence, we propose that fertility of transgenic tobacco in this study could be due to the absence of a biosynthetic product in the anthers of these transgenic tobacco plants that could trigger male sterility as proposed by Flavell (1974) (Schnable and Wise 1998).

Methomyl sensitivity assay for these transgenic plants was carried out since it was shown that expression of URF13 in many heterologous systems like *E. coli*, yeast, plants (rice, tobacco) has rendered them sensitive to methomyl treatment (Braun et al. 1989; Braun et al. 1990; Chaumont et al. 1995; Glab et al. 1993; Huang et al. 1990; Korth and Levings 1993; Sridhar 2001; von Allmen et al. 1991). The interaction of methomyl with URF13 protein expressed in anthers of these transgenic tobacco plants would have resulted in cell death (i.e.) tapetal destruction leading to male sterility, as cell death was observed with the transgenic tobacco carrying pCAT121 construct in this study (Fig. 4). The production of viable pollen and seeds in the methomyl-treated transgenic plants (carrying p500 and p755 construct) could be due to either non-availability of methomyl in the anthers during URF13 expression or degradation of methomyl within the plants (DuPont technical manual). Since the methomyl treatment was done on whole flowers methomyl has to pass through several layers (sepal, petal, etc) before it can reach tapetum (innermost layer of anthers) where URF13 protein is expressed. Methomyl might have got degraded before reaching tapetum thereby resulting in fertile flowers (viable pollen and seeds production) (DuPont technical manual). Another possibility could be that the analysed transgenic plants had low levels of URF13 expression; large number of transgenic plants has to be analysed to come to any conclusion. One strategy to obtain controllable male sterility using T-*urf*13 gene driven by anther-specific promoter would be to co-express an analogue of methomyl in the anthers of those plants or develop a delivery system to introduce methomyl directly to tapetum of these plants.

In summary, the T-*urf*13 gene in maize was effective in generation of male sterile plants and constitutive expression of this gene in other plants like tobacco and rice has been shown to result in methomyl sensitivity, but not male sterility. Hence, this study was done to test whether expression of T-*urf*13 gene in anthers would result in male sterility by itself or male sterility can be induced by methomyl treatment with a possible application in hybrid seed production. This is the first attempt to express T-*urf*13 gene under anther-specific promoter (TA29) in plants. These transgenic tobacco plants carrying T-*urf*13 gene under anther-specific promoter (p500 and p755) were fertile except for one plant (500-20). The fertility observed in these transgenic plants could be due either to low levels of expression of T-*urf*13 gene or absence of a biosynthetic product in the anthers of these plants that could mimic methomyl or T-toxin to trigger male sterility. The transgenic plants (carrying p755 and p500 constructs) treated with methomyl produced pollen and seeds. This may be due to non-availability of methomyl for the URF13 protein to interact and result in tapetal degeneration and possibly male sterility, even though cell death was observed with methomyl treatment in transgenic tobacco plants expressing URF13 constitutively (pCAT121 construct). Many transgenic plants have to be analysed and expression levels of URF13 in the anthers of those plants also have to be ascertained before any conclusions can be made.

## Abbreviations

PCR: Polymerase chain reaction
DNA: Deoxyribonucleic acid
SDS: Sodium dodecyl sulphate
CMS: Cytoplasmic male sterility
*rrn*26: Ribosomal RNA 26
MS: Murashige and Skoog
